# The Differential Effect of Cannabidiol on the Composition and Physicochemical Properties of Keratinocyte and Fibroblast Membranes from Psoriatic Patients and Healthy People

**DOI:** 10.3390/membranes11020111

**Published:** 2021-02-04

**Authors:** Barbara Szachowicz-Petelska, Wojciech Łuczaj, Adam Wroński, Anna Jastrząb, Izabela Dobrzyńska

**Affiliations:** 1 Faculty of Chemistry, University in Białystok, Ciołkowskiego 1K, 15-245 Białystok, Poland; basia@uwb.edu.pl; 2 Department of Analytical Chemistry, Medical University of Białystok, Mickiewicza 2d, 15-222 Białystok, Poland; wojciech.luczaj@umb.edu.pl (W.Ł.); anna.jastrzab@umb.edu.pl (A.J.); 3 Dermatological Specialized Center ”DERMAL” NZOZ in Białystok, Nowy Swiat 17/5, 15-453 Białystok, Poland; adam.wronski@dermal.pl

**Keywords:** psoriasis, cannabidiol, UV radiation, fibroblasts, keratinocytes, electrical properties, phospholipids, sialic acid, malondialdehyde

## Abstract

The development of psoriasis is accompanied by oxidative stress, which can modify the components of skin cells. Therefore, the aim of this study was to evaluate the effect of cannabidiol (CBD), an antioxidant and anti-inflammatory phytocannabinoid, on the composition and physicochemical properties of the membranes of healthy and psoriatic keratinocytes and fibroblasts exposed to ultraviolet A (UVA) and ultraviolet B (UVB) radiation. In psoriasis-altered cells, decreased levels of the main groups of phospholipids and increased levels of sialic acid and malondialdehyde (MDA), a lipid peroxidation product, as well as negative charge of cell membranes compared to non-diseased cells, were found. On the other hand, UVA/B radiation increased the levels of phospholipids and MDA in both groups of cells. Moreover, psoriatic cells were characterized by lower levels of sialic acid and negative charge of cell membranes, while non-diseased cells showed the opposite response. The CBD treatment intensified some of the changes (phospholipid content and membrane charge) caused by the radiation of psoriatic cells, while it prevented these changes in the cells of healthy people. The results of this study indicate that CBD can prevent structural and functional changes to the membranes of healthy skin cells during phototherapy for psoriasis.

## 1. Introduction

The cell membrane is a semipermeable structure that separates the interior of the cell from the external environment and is an essential element for the proper functioning of individual cells as well as tissues and the entire organism. The structure of the cell membrane is based on the lipid bilayer, and its phospholipids play an important role in modulating the immune response and signal transduction [1]. In addition, the cell membrane includes other lipids, proteins, and carbohydrates, and has various roles in transport, intercellular signaling, and receptor function. Furthermore, the cell membrane provides the cell with integrity as well as active contact with the environment [2].

Under pathophysiological conditions, changes in the composition of phospholipids and proteins of cell membranes occur. As a result, there are changes in the properties of the lipid bilayer and thus the electric charge of the membrane, which, especially in relation to skin cells, may also be altered under the influence of exogenous physical and chemical factors. Modifications to the electrical properties of the membrane also affect the balance between the elements of the membrane and its surroundings. Therefore, the assessment of the surface charge density of a cell membrane depending on the pH and the parameters describing the membrane, such as the surface concentrations of acidic groups (*C_TA_*) and basic groups (*C_TB_*) and their association constants with hydrogen ions (*K_AH_*) and hydroxyl ions (*K_BOH_*) ions, allows one to monitor the pathophysiological changes resulting from the development of disease and the influence of both physical and chemical factors on tissues and cells [3,4].

The above relationships also apply to skin cells during the development of diseases, such as atopic dermatitis and psoriasis, and their local pharmacotherapy as well as pharmacotherapy combined with phototherapy using ultraviolet UVA and UVB radiation [5,6]. UVA radiation penetrates deeply through the epidermis into the dermis, affecting the metabolism of both keratinocytes (the main cell type of the epidermis) and fibroblasts (the main cell type of the dermis). On the other hand, UVB radiation mainly affects epidermal cells [7]. Both types of radiation, differing in energy and depth of skin penetration, cause different biological effects; but both UVA and UVB radiation intensify the generation of reactive oxygen species (ROS) in cells. Consequently, ROS can induce oxidative modifications of proteins, DNA, and lipids, thus generating reactive endogenous electrophiles [8].

During the development of psoriasis, inflammation occurs and cell redox homeostasis is disturbed; these conditions play a key role in pathophysiological processes, especially those of skin cells [1,9]. Additionally, both the pharmacotherapy used in the treatment of psoriasis and phototherapy with the use of UV radiation intensify oxidative stress. It has been reported, inter alia, that in the case of skin treatment with psoralen assisted by UVA radiation, there is a massive production of singlet oxygen in the skin [10]. On the other hand, the use of phototherapy with UVB radiation causes local intensification of the redox imbalance with a simultaneous anti-inflammatory effect, which is probably the result of the activation of antiproliferative and proapoptotic pathways [11]. Therefore, it seems justified to look for substances, mainly among natural compounds, that can reduce oxidative stress and its consequences by regulating the redox balance.

Cannabidiol (CBD), a pharmacologically active phytocannabinoid found in *Cannabis sativa* L., exhibits a wide range of biological activities, including antioxidant and anti-inflammatory effects, among others, but it has no psychoactive effect [5,12,13]. CBD regulates the redox state of cells by preventing the formation of ROS, increasing the level/activity of nonenzymatic and enzymatic endogenous antioxidants at the transcriptional level, activating the transcription factor nuclear factor erythroid 2-related factor 2, and directly protecting against functioning antioxidants [5,14,15]. The most noticeable antioxidant effects of CBD, a lipophilic compound, are its localization in cell membranes and its prevention of oxidative modifications of lipids and proteins that are components of the cell membrane [5,16]. It has been found that the use of CBD reduces lipid peroxidation, which is assessed by measuring the level of α,β-unsaturated aldehydes such as malondialdehyde (MDA) and 4-hydroxynonenal [5,14,16]. In addition, it decreases the formation of adducts of these aldehydes with proteins [5,16] and prevents the modification of cell transport proteins in the skin [5,14,16]. Furthermore, by protecting lipids and proteins against oxidative damage, CBD prevents changes in cell signaling [14,17].

Therefore, the aim of this study was to determine the effects of CBD on the composition as well as the electrical properties of the cell membranes of keratinocytes and fibroblasts irradiated with UVA/B radiation from psoriatic patients and healthy people.

## 2. Materials and Methods

### 2.1. Primary Cell Isolation

Skin tissues were collected from six untreated patients diagnosed with psoriasis vulgaris (3 men and 3 women; age range: 26–53 years old, mean age: 40 years old; these patients were randomly selected from a cohort of 25 patients) and six healthy volunteers (sex- and age-matched individuals forming a control group; age range: 26–58 years old, mean age: 41 years old). Patients qualified as having psoriasis vulgaris were those whose lesions affected at least 10% of their total body surface for at least 6 months. Psoriasis severity was assessed using the psoriasis index and severity index (range: 14–25; median: 18). The major exclusion criteria for both groups were as follows: Receiving topical or oral medications during the 4 weeks before the study, comorbidities, or smoking/alcohol abuse. All participants gave their informed consent for inclusion in this research. This research was carried out in accordance with the Helsinki Declaration, and the protocol was approved by the Local Bioethics Commission at the Medical University of Białystok (Poland), No. R-I-002/289/2017. 

Skin fragments were taken for histopathological examination (hematoxylin-eosin staining). The remaining samples were washed with phosphate-buffered saline (PBS), 50 U/mL penicillin, and 50 μg/mL streptomycin, and incubated overnight in 1 mg/mL dispase at 4 °C to separate the epidermis from the dermis. After incubation, the epidermis was digested for 20 min at 37 °C using trypsin/EDTA, and the separated keratinocytes were washed and resuspended in PBS containing a protease inhibitor mixture.

The dermis was sliced and placed into culture plates containing fibroblast culture medium consisting of Dulbecco’s modified Eagle medium, 10% fetal bovine serum, 50 U/mL penicillin, and 50 μg/mL streptomycin. Samples were incubated in a humidified atmosphere of 5% CO_2_ at 37 °C until the fibroblasts emigrating from the slices reached 80% confluence.

### 2.2. Cell Culture and Treatment

The keratinocytes and fibroblasts were washed with PBS (37 °C) and subjected to UVA (365 nm; 30 J/cm^2^) and UVB (312 nm; 60 mJ/cm^2^) irradiation with a Bio-Link Crosslinker (BLX 312/365; Vilber Lourmat, Germany) in cold PBS (4°C). This temperature was selected to avoid heat stress and oxidation of the medium components. The radiation doses were selected to ensure 70% cell viability. After irradiation, the medium was changed to one containing 4 µM CBD (dissolved in ethanol, of which the final concentration in the medium was 0.1%). CBD was purchased from Sigma-Aldrich (St. Louis, MO, USA). This concentration of CBD did not alter the morphology or proliferation of keratinocytes [18,19], and the cell viability was measured by the 3-(4,5-dimethylthiazol-2-yl)-2,5-diphenyltetrazolium bromide assay [20].

Ultimately, the keratinocytes and fibroblasts were divided into two main experimental groups according to the origin of the cells: Group 1, cells derived from the skin of healthy people; and group 2, cells derived from the skin of psoriatic patients.

Group set 1

The cells in group set 1 were subdivided into six subgroups and treated as follows:

- Control group (Ctr), keratinocytes or fibroblasts from the skin of healthy subjects were cultured in standard medium;
- CBD group (CBD), keratinocytes or fibroblasts from the skin of healthy subjects were cultured for 24 h in medium containing 4 µM CBD;
- UVA group (UVA), keratinocytes or fibroblasts from the skin of healthy subjects were exposed to UVA radiation (30 J/cm^2^);
- UVB group (UVB), keratinocytes or fibroblasts from the skin of healthy subjects were exposed to UVB radiation (60 mJ/cm^2^);
- UVA + CBD group (UVA + CBD), keratinocytes or fibroblasts from the skin of healthy subjects were exposed to UVA irradiation (30 J/cm^2^) and then cultured for 24 h in medium containing 4 µM CBD;
- UVB + CBD group (UVB + CBD), keratinocytes or fibroblasts from the skin of healthy subjects were exposed to UVB irradiation (60 mJ/cm^2^) and then cultured for 24 h in medium containing 4 µM CBD.

Group set 2

The cells in group set 2 were subdivided into six subgroups and treated as follows:

- Psoriasis group (Ps), keratinocytes or fibroblasts from the skin of psoriatic patients were cultured in standard medium;
- Ps + CBD group (Ps + CBD), keratinocytes or fibroblasts from the skin of psoriatic patients were cultured for 24 h in medium containing 4 µM CBD;
- Ps + UVA group (Ps + UVA), keratinocytes or fibroblasts from the skin of psoriatic patients were exposed to UVA radiation (30 J/cm^2^);
- Ps + UVB group (Ps + UVB), keratinocytes or fibroblasts from the skin of psoriatic patients were exposed to UVB radiation (60 mJ/cm^2^);
- Ps + UVA + CBD group (Ps + UVA + CBD), keratinocytes or fibroblasts from the skin of psoriatic patients were exposed to UVA radiation (30 J/cm^2^) and cultured for 24 h in medium containing 4 µM CBD;
- Ps + UVB + CBD group (Ps + UVB + CBD), keratinocytes or fibroblasts from the skin of psoriatic patients were exposed to UVB radiation (60 mJ/cm^2^) and cultured for 24 h in medium containing 4 µM CBD.

Finally, the keratinocytes and fibroblasts from the above groups were washed with PBS and used in further examinations.

### 2.3. Isolation and Analysis of Phospholipids

According to the Folch method, total phospholipids were extracted [21]. To the skin cell membranes of keratinocytes or fibroblasts, a 1:2 chloroform/methanol mixture was added. The mixture was vortexed well and then an additional volume of chloroform, followed by Milli-Q water, was added. In all steps, the mixtures were strongly vortexed. Finally, the samples were centrifuged at 4000× *g* for 20 min at room temperature to obtain a two-phase system (an aqueous upper phase and an organic lower phase). After drying, the total phospholipid extract from the organic phase was resuspended in 200 μL of 1:2 chloroform/methanol. Separation of the phospholipid classes was achieved using normal-phase high-performance liquid chromatography (HPLC) with a silica gel column. A 95.2:3.7:1.1 (*v/v/v*) acetonitrile/methanol/85% phosphoric acid mixture was used as the eluent with isocratic elution at a flow rate of 1 mL/s and a detection wavelength of 214 nm [22].

**Figure 1:**
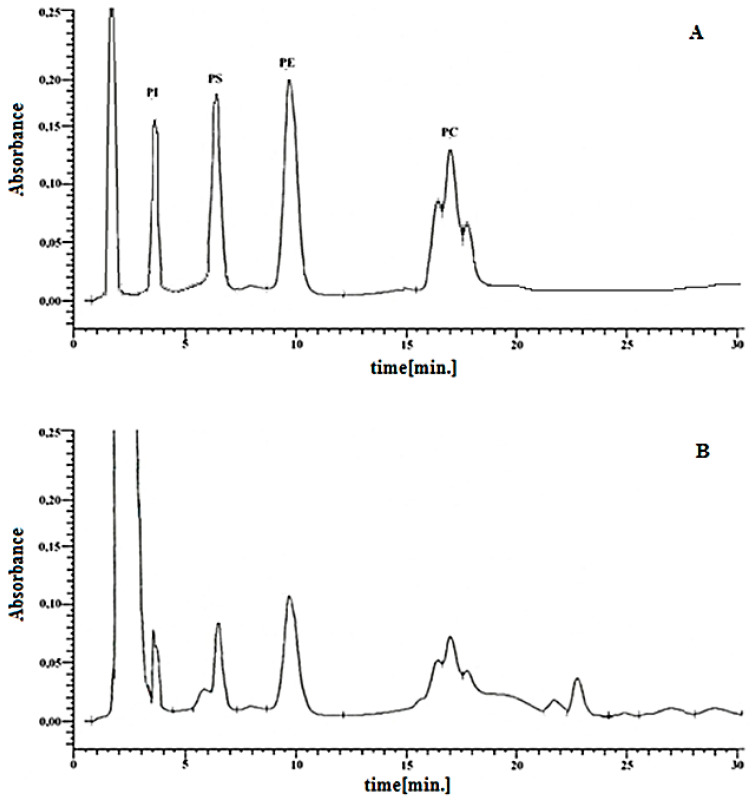
Chromatogram of the phospholipid standard mixture (**A**) and total keratinocyte lipid extract (**B**) PI-phosphatidylinositol, PS-phosphatidylserine, PE-phosphatidylethanolamine, PC-phosphatidylcholine.

An example chromatogram of four phospholipid classes obtained from keratinocytes of healthy subjects is shown in Figure 1. Phospholipid classes were identified on the basis of the retention times of their standards: Phosphatidylinositol—t_PI_ = 3.52 min; phosphatidylserine—t_PS_ = 6.48 min; phosphatidylethanolamine—t_PE_ = 9.65 min; and phosphatidylcholine—t_PC_ = 16.35 min, 16.93 min, 17.98 min. Quantification of each phospholipid class was conducted using calibration curves constructed for corresponding phospholipid standards in the concentration range from 5 µg/mL to 100 µg/mL. PI (phosphatidylinositols, soy), PS (L-λ-phosphatidylserines, soy), PE (phosphatidylethanolamine, soy), and PC (phosphatidylcholines, egg) were purchased from Cayman Chemicals (Ann Arbor, MI, USA) and used in our study as phospholipid standards. The correlation coefficients (R^2^) of the obtained curves were 0.9994, 0.9991, 0.9988, and 0.9996 for PI, PS, PE, and PC, respectively. Moreover, to eliminate the matrix effect, all working solutions of standards used for calibration curves were obtained by adding an appropriate amount of each PL standard to control keratinocytes before lipid extraction. The limit of quantification (LOQ; µg/mL) was 4.61, 4.65, 4.53, and 4.32 for PI, PS, PE, and PC, respectively, while the corresponding limit of detection (LOD; µg/mL) was 1.52, 1.51, 1.49, and 1.42. 

### 2.4. Determination of the Sialic Acid Level

The modified Svennerholm’s resorcinol method was used to determine the total sialic acid content in the keratinocyte and fibroblast membranes [23]. Using a diode array spectrophotometer (Hewlett Packard), the color intensity at 630 was measured. The sialic acid concentration was found based on the standard curve of the N-acetylneuraminic acid solution.

### 2.5. Estimation of Lipid Peroxidation

By measuring the MDA level, lipid peroxidation was estimated. Aldehyde level was measured by GC/MSMS, as the O-PFB-oxime-TMS derivative, using a modified method of Luo et al. [24]. Benzaldehyde-D6, as an internal standard, was added to the cell lysates, and the aldehydes were derivatized by the addition of O-(2,3,4,5,6-pentafluoro-benzyl) hydroxyamine hydrochloride (0.05 M in PIPES buffer, 200 µL) and incubated for 60 min at room temperature. Derivatized aldehydes were analyzed using a 7890A GC–7000 quadrupole MS/MS (Agilent Technologies, Santa Clara, CA, USA) equipped with a HP-5ms capillary column (0.25 mm internal diameter, 0.25 µm film thickness, 30 m length) and detected in selected ion monitoring mode. The ions used were as follows: *m*/*z* 204.0 and 178.0 for MDA-PFB, and *m/z* 307.0 for the internal standard derivative [3,25].

### 2.6. Electrochemical Methods

Using a Zetasizer Nano ZS apparatus (Malvern Instruments, Malvern, UK) the electrophoretic mobility (u) and zeta potential (ξ) of the cell membranes were measured, as presented earlier [26]. Then, the surface charge density was determined from the electrophoretic mobility using the following formula:

(1) $$\delta =\frac{\eta \cdot u}{d}$$

where *u* is the electrophoresis mobility, *η* is the viscosity of the solution, and *d* is the diffuse layer thickness. The diffuse layer thickness was determined from the formula $d=\sqrt{\frac{\varepsilon \cdot \varepsilon_{0}\cdot R\cdot T}{2\cdot F^{2}\cdot I}}$ where *ε*·*ε*_0_ is the permeability of the electric medium, *R* is the gas constant, *T* is the temperature, *F* is the Faraday constant (96,487 [C∙mol^−1^]), and I is the ionic strength of 0.9% NaCl.

The dependence between the surface charge density of the cell membranes of skin cells and the pH of the electrolyte solution is described using the mathematical equations provided by Dobrzyńska et al. [27]. This model assumes the existence of four equilibria between the functional groups on the membrane surface and the H^+^, OH^−^, Na^+^, and Cl^−^ ions. The final equation describing the surface charge density of the membrane (δ) is presented down below:

(2) $$\frac{\delta }{F}=\frac{C_{TB}\cdot a_{H^{+}}}{a_{H^{+}}\left(1+K_{BCl}\cdot a_{Cl^{-}}\right)+\;K_{BOH}\cdot K_{w}}-\;\frac{C_{TA}}{K_{AH}\cdot a_{H^{+}}+\;K_{ANa}\cdot a_{Na^{+}}+1\;}$$

where *C_TA_* is the total surface concentration of the acidic groups, *C_TB_* is the total surface concentration of the basic groups, and *K_AH_, K_ANa_*, *K_BOH_*, and *K_BCl_* are the association constants.

By introducing the results of the electric charge dependence of the cell membrane as a function of pH to the theoretical equations that we derived to describe the charge dependence on the solution composition, we determined the parameters characterizing the membrane such as the total concentrations *C_TA_* and *C_TB_* and their association constants *K_AH_* and *K_BOH_*.

### 2.7. Statistical Analysis

The data obtained in this study were expressed as the mean ± standard deviation. The data were analyzed by Kruskal–Wallis test with post hoc Dunn’s multiple comparisons tests for multiple comparisons to identify significant differences between groups. *p* values < 0.05 were considered significant. Statistical analyzes were performed using GraphPad Prism for Windows version 7.0.0 (GraphPad software, San Diego, CA, USA).

## 3. Results

Due to the small number of samples (*n* = 6), we have used nonparametric methods, which are recommended in such a case. Obtained data were analyzed by Kruskal–Wallis test with post hoc Dunn’s multiple comparisons tests to identify significant differences between groups. The results of this study showed that the development of psoriasis caused changes in the chemical composition of the cell membranes of epidermal cells (keratinocytes) and dermal cells (fibroblasts) compared to those from healthy individuals. In addition, treatment with CBD and/or UV radiation resulted in additional modifications.

It was found that the development of psoriasis led to a reduction in the levels of individual types of phospholipids, including phosphatidylcholine (PC), phosphatidylethanolamine (PE), phosphatidylserine (PS), and phosphatidylinositol (PI), in keratinocytes and fibroblasts from psoriatic patients compared to cells from healthy individuals (Table 1 and Table 2). Exposing the skin cells from healthy people and psoriatic patients to CBD reduced the levels of all types of phospholipids. After the UVA/B irradiation of skin cells from both healthy people and psoriatic patients, the phospholipid levels in the individual groups increased. Moreover, the skin cells treated with CBD after irradiation with UVA or UVB rays from healthy people were characterized by a significant reduction in the phospholipid levels compared to the irradiated cells without CBD treatment and had levels similar to the control values; while in the case of cells from psoriatic patients, a further increase in the phospholipid levels was observed.

**Figure 2:**
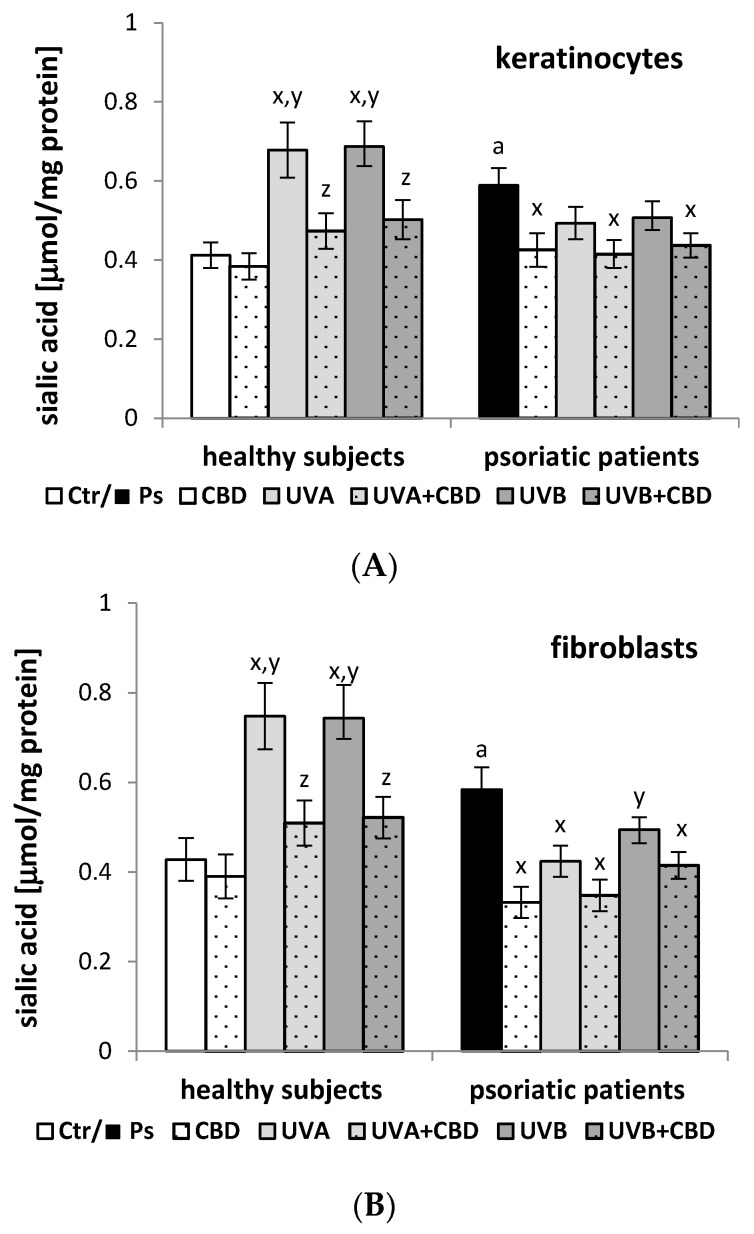
The sialic acid content in keratinocytes (**A**) and fibroblasts (**B**) isolated from skin of healthy subjects (Ctr) (*n* = 6) and psoriatic patients (Ps) (*n* = 6) exposed to UVA (30 J/cm^2^) and UVB (60 mJ/cm^2^) radiation as well as untreated and treated with cannabidiol (CBD) (4 μM). The mean values ± SD and statistically significant differences for *p* < 0.05 are presented.; ^a^—differences between Ps and Ctr groups; ^x^—differences vs. Ctr or Ps group; ^y^—differences vs. CBD treated groups [CBD and Ps + CBD]; ^z^—differences vs. UVA or UVB treated groups [UVA/UVB and Ps + UVA/UVB].

Furthermore, in addition to the changes in the composition of the cell membrane phospholipids, it was shown that the development of psoriasis led to an increase in sialic acid levels in both keratinocytes and skin fibroblasts (by about 70% and 30%, respectively) compared to control cells (Figure 2). In contrast, treatment with CBD significantly reduced the level of sialic acid, but only in the cells from psoriatic patients, approaching the level observed in the cells from healthy people. The keratinocytes and fibroblasts exposed to UVA or UVB radiation from healthy people showed higher levels of sialic acid than the control cells, and the cells from the psoriatic patients gave the opposite response. On the other hand, the administration of CBD after UVA/B irradiation decreased the level of sialic acid in both keratinocytes and fibroblasts from healthy people and psoriatic patients, compared to irradiated cells without CBD treatment. However, greater changes were observed in the cells derived from healthy people.

**Figure 3:**
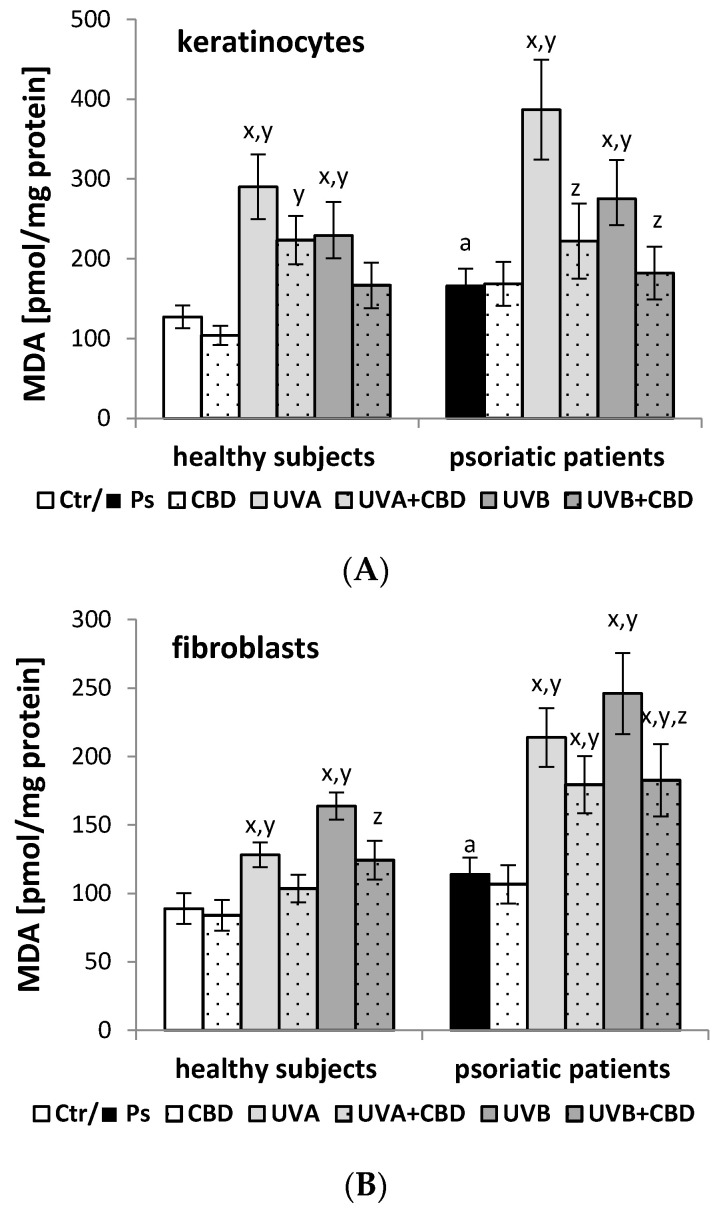
The level of lipid peroxidation product [malondialdehyde (MDA)] in keratinocytes (**A**) and fibroblasts (**B**) isolated from skin of healthy subjects (Ctr) (*n* = 6) and psoriatic patients (Ps) (*n* = 6) exposed to UVA (30 J/cm^2^) and UVB (60 mJ/cm^2^) radiation as well as untreated and treated with CBD (4 μM). The mean values ± SD and statistically significant differences for *p* < 0.05 are presented; ^a^—differences between Ps and Ctr groups; ^x^—differences vs. Ctr or Ps group; ^y^—differences vs. CBD treated groups [CBD and Ps+CBD]; ^z^—differences vs. UVA or UVB treated groups [UVA/UVB and Ps + UVA/UVB].

Both the development of psoriasis and the UV irradiation of skin cells are accompanied by a redox equilibrium shift towards oxidative conditions, the consequence of which is an increase in the level of the lipid peroxidation product MDA in keratinocytes and fibroblasts of patients with psoriasis (Figure 3). Both UVA and UVB radiation increased the MDA level in skin cells from healthy people and psoriatic patients. The administration of CBD after UVA/B irradiation decreased the MDA level in both keratinocytes and fibroblasts from healthy people and psoriatic patients in comparison to irradiated cells without CBD treatment. The changes observed in keratinocytes from psoriatic patients were more pronounced. However, the CBD treatment of keratinocytes and fibroblasts from both healthy people and psoriatic patients did not cause any statistically significant changes.

**Figure 4:**
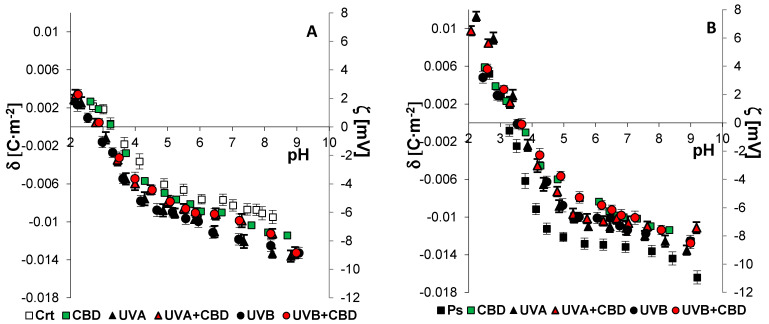
The surface charge density (**left axis**) and corresponding zeta potential (**right axis**) of keratinocytes isolated from skin of healthy subjects (Ctr) (**A**) (*n* = 6) and psoriatic patients (Ps) (**B**) (*n* = 6) exposed to UVA (30 J/cm^2^) and UVB (60 mJ/cm^2^) radiation as well as untreated and treated with CBD (4 μM). The mean values ± SD are presented.

**Figure 5:**
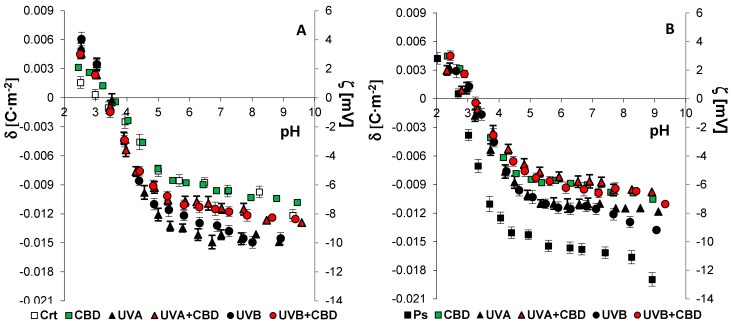
The surface charge density (**left axis**) and corresponding zeta potential (**right axis**) of fibroblasts isolated from skin of healthy subjects (Ctr) (**A**) (*n* = 6) and psoriatic patients (Ps) (**B**) (*n* = 6) exposed to UVA (30 J/cm^2^) and UVB (60 mJ/cm^2^) radiation as well as untreated and treated with CBD (4 μM). The mean values ± SD are presented.

Figure 4 and Figure 5 present the surface charge density of the cell membranes of keratinocytes and fibroblasts (or the corresponding zeta potential), respectively, as a function of pH. The values of *C_TA_, C_TB_, K_AH_*, and *K_BOH_* were calculated using theoretical equations describing the adsorption of ions in the solution on the surface of the cell membrane (described in the Methods section). The constants determined after substitution into Equation (2) provided theoretical values of the surface charge density, which were consistent with the experimental values.

In the cell membranes of keratinocytes and fibroblasts from psoriatic patients, an increase in the negative electric charge was observed compared to the control, corresponding to an increase in the *C_TA_*, *C_TB_*, and *K_BOH_* values, and a decrease in *K_AH_* (Table 3). The changes in the composition of the phospholipids of the cell membrane may result from the appearance of new ones or the disappearance of already existing groups during the modifications taking place during the development of psoriasis.

The administration of CBD significantly reduced the negative membrane charge, *C_TA_*, and *K_BOH_*, as well as increased the *K_AH_* value of skin cells from psoriatic patients. After irradiating the skin cells from healthy people with UVA/B radiation, the negative electric charge of the membrane, *C_TA_*, *C_TB_*, and *K_BOH_* increased significantly, while *K_AH_* decreased compared to the values of the control cells. In contrast, in the case of patient-derived cells, UV radiation reduced the negative charge of the membrane, *C_TA_*, and *K_BOH_* and increased *K_AH_* compared to the psoriasis group. The keratinocytes and fibroblasts exposed to CBD after UVA/B irradiation from healthy people were characterized by a significantly reduced negative membrane charge, *C_TA_*, *C_TB_*, and *K_BOH_*, and an increase in *K_AH_* compared to the values for irradiated cells without CBD treatment. Interestingly, there were statistically significant changes in all parameters characterizing the membrane only in fibroblasts treated with CBD after UVA/B irradiation from psoriatic patients, compared to cells from psoriatic patients only exposed to UV irradiation.

## 4. Discussion

The development of psoriasis is accompanied by systemic and local oxidative stress, and disturbed redox homeostasis plays a key role in pathophysiological processes, particularly in skin cells [9,28]. The overproduction of ROS favors their reactions with cell components, especially cell membrane phospholipids containing polyunsaturated fatty acids, with the formation of, among others, α,β-unsaturated aldehydes, which together with ROS modify the structures of other components of biological membranes [9,29]. This causes changes in the composition of cell membranes and, as a consequence, their physicochemical properties, including electrical ones, which may lead to changes in the functions of the cell membranes and the cells they protect [26,30].

The results of this study demonstrated that during the development of psoriasis, the levels and localization of the cell membrane phospholipids decreased and the levels of sialic acid and the lipid peroxidation product MDA increased, thus causing an increase in the negative charge on the surface of keratinocyte and fibroblast membranes. It is known that such changes may affect the interactions between cells as well as between cells (especially cell membranes) and ions or molecules in their environment. As a result, they can cause cell function disorders and promote disease development [31,32,33].

Accordingly, various forms of therapy for psoriasis are used, including phototherapy with the use of UV radiation as the main or additional factor in therapy [34]. However, it has been shown previously that UV radiation intensifies oxidative stress in skin cells, especially pathologically changed cells, and this finding was confirmed by the results of this study [5,8,14,35]. Slight oxidative stress may have a positive effect on cellular metabolism [36]; however, if oxidative stress is intensified, it may increase pathophysiological changes in skin cells [8,37]. As a consequence of UVB radiation, changes in the metabolism of skin cell membrane components such as phospholipids and proteins have been observed [5,14,37,38]. On the other hand, UVA radiation causes a significant increase in the activity of membrane transporters, e.g., bilitranslocase [25].

The results of this study showed that both UVA and UVB radiation increased the levels of different classes of phospholipids (PC, PE, PS, and PI) in keratinocytes and fibroblasts from both psoriasis patients and healthy individuals. Similarly, literature data demonstrate that fibroblasts exposed to UVA and UVB radiation as well as keratinocytes exposed to UVB radiation have increased PC and PE levels and decreased sphingomyelin (SM) levels [25,33,39,40]. The increase in the PC level and the decrease in the SM level may be related to the activation of sphingomyelinase, which hydrolyzes SM to ceramides and phosphocholine, a compound that mediates the synthesis of PC and may lead to its increase [41]. Ceramides, on the other hand, play the role of secondary messengers in the cell signaling pathways of inflammation, oxidative stress, and apoptosis, as well as in all metabolic and pathophysiological pathways modulated by these states [42]. Meanwhile, an increase in the level of glycerophospholipids caused by UV radiation may also indicate an increased activity of enzymes such as choline kinase or ethanolamine kinase as well as enzymes participating in the biosynthesis of these lipids, which has been observed in other diseases [43]. In this study, greater changes in the phospholipid levels after UV radiation were observed in healthy people than in psoriasis patients. This may be due to the fact that a significant increase in lipid peroxidation as assessed by the MDA level was observed in keratinocytes and fibroblasts of psoriasis patients, similar to the findings of previous studies [44,45]. Although UV radiation is one of the factors that alters phospholipid metabolism, our results revealed that exposure of keratinocytes and fibroblasts to UV radiation from psoriasis patients activated adaptive mechanisms that shifted the phospholipid levels towards the levels seen in the cells from healthy subjects, indicating a positive aspect of phototherapy.

Regardless of the qualitative and quantitative changes in the structures of phospholipids under the influence of either UVA or UVB radiation, the total level of sialic acid in the membranes of keratinocytes and fibroblasts from healthy people increased in this study. Literature data confirm the relationship between an increase in sialic acid levels and sialylation enhanced by UV [46], which plays an important role in cell signaling [47] and cell adhesion [48]. In contrast, in the case of keratinocytes and fibroblasts exposed to UV radiation from psoriasis patients, the level of sialic acid decreased, which may be related to the induction of apoptosis in response to UV radiation [49]. Literature data show that early apoptosis is accompanied by a decrease in the amount of sialic acid in the cell membrane [50]. A lower level of this acid also indicates a reduction in inflammation, since sialic acid is considered a marker of inflammation in the body [51,52].

Alterations in both the phospholipid structure and the level of sialic acid, which is a component of glycolipids and glycoproteins in the membranes of keratinocytes and fibroblasts, lead to a change in the number of functional groups on the surface of the cell membrane. Consequently, these changes alter the electrical properties of the cell membrane, which was confirmed by our results. In the case of cells from healthy people, both UVA and UVB radiation, respectively, caused an increase in the negative charge on the surface of the tested cells compared to the control cells. Meanwhile, in the case of cells from psoriasis patients, there was a decrease in the negative charge on the cell membrane. Modifications of the composition and electric charge of cell membranes affect, inter alia, changes in membrane permeability and functions of skin cells, including receptor functions and signaling. In addition, literature data show that the interactions between keratinocytes and fibroblasts, as well as these cells with the basement membrane, are crucial for maintaining homeostasis in the skin [53,54].

Due to the above changes, in the case of both the development of psoriasis and phototherapy, there is a need to search for new or modified therapies that are more effective. Therefore, CBD, an antioxidant and anti-inflammatory phytocannabinoid, has been used as a potential therapeutic compound [55]. Previous studies have shown that CBD penetrates into keratinocytes and accumulates mainly in the cell membrane [14]; this process is believed to be a consequence of the lipophilic nature of CBD [56]. Thus, since CBD can penetrate and accumulate in the membranes of skin cells, it can actively participate in modeling the effects of oxidative stress on membrane components [14]. In this study, the treatment of keratinocytes and fibroblasts from psoriasis patients with CBD led to a further reduction in the levels of all assessed classes of membrane phospholipids, including PC, PE, PS, and PI, which are lowered during the development of psoriasis. Therefore, this decrease can be considered a continuation of the proapoptotic changes observed in these cells during the development of psoriasis. The direction of these changes is consistent with the latest reports indicating that CBD may increase oxidative stress in keratinocytes from patients with psoriasis [5]. Lipid peroxidation disturbs the asymmetry of membrane lipids, transferring PS to the outer surface of the membrane [57]. The translocation of phospholipids in cell membranes, especially PS, has been recognized as one of the most important markers of the initial phase of apoptosis and is a key signal that triggers phagocytosis in both apoptotic and necrotic cells [58,59]. In addition, the results of our earlier work have shown that CBD treatment of keratinocytes from psoriasis patients causes an increase in SM levels [33]. This finding indicates a correlation between a decrease in PC levels and an increase in SM levels with a reduction in the negative charge of both the epidermis (keratinocytes) and dermis (fibroblasts), which is suggested by the fact that the association constant of negatively charged groups in SM with H^+^ ions has a greater value than the association constant of these groups in PC [60,61].

Moreover, we observed that the exposure of skin cells irradiated with UV to CBD significantly prevented the changes induced by this radiation only in the case of cells obtained from healthy people. This applies to both structural changes, including the levels of phospholipids, sialic acid, and lipid peroxidation products, as well as functional changes manifested by a decrease in the negative charge on the cell membrane surface, which, as shown by the results of this study, was caused by a reduction in the sialic acid content. These changes were accompanied by a decrease in the values of *C_TA_*, *C_TB_*, and *K_BOH_*, as well as an increase in the value of *K_AH_* on the membrane surface. Changes in the values of these constants suggest that the negatively charged groups appearing on the surface show weaker acidic properties, i.e., the same as in healthy people. On the other hand, in the case of cells from psoriatic patients, the effect of CBD caused an intensification of changes in the phospholipid content and physicochemical properties, including electrical properties, of the membrane, thus indicating a return to the control values.

## 5. Conclusions

The obtained results confirm the antioxidant effect of CBD in relation to healthy cells and cells from psoriasis patients because, like other antioxidants, CBD reduces the severity of lipid peroxidation, as assessed by the MDA level. However, the results of this study suggest that both under the influence of UV radiation and CBD, metabolic changes occur in keratinocytes and fibroblasts from both healthy people and psoriasis patients, thus promoting modification of the cell membrane composition. The consequence of this altered composition is that the physicochemical properties of these cell membranes are changed, with greater changes being observed in the membranes of skin cells from psoriasis patients, which may favor the apoptosis of these cells. In contrast, the exposure of skin cells from healthy people to UV and CBD significantly prevented the changes caused by this radiation, suggesting that CBD, as a component of phototherapy, has a beneficial effect on unchanged skin for the treatment of psoriasis. In the context of the obtained results pointing to the beneficial effect of CBD on skin cells, it would be interesting to assess the impact of natural phytocannabinoids present in *Cannabis sativa* [62]. Therefore, potential synergistic action of these cannabinoids should be confirmed or not in the future studies.

## Figures and Tables

**Table 1:** Phospholipids membrane composition of keratinocytes isolated from skin of healthy subjects (Ctr) (*n* = 6) and psoriatic patients (Ps) (*n* = 6) exposed to ultraviolet A (UVA) (30 J/cm^2^) and ultraviolet B (UVB) (60 mJ/cm^2^) radiation as well as untreated and treated with cannabidiol (CBD) (4 μM). The mean values ± SD and statistically significant differences for *p* < 0.05 are presented.

Groups	PI	PS	PE	PC
	µg/mg Protein			
**Ctr**	**1.14 ± 0.07**	**1.65 ± 0.09**	**3.41 ± 0.32**	**4.01 ± 0.14**
**CBD**	0.82 ± 0.09	1.21 ± 0.07	2.81 ± 0.16	3.28 ± 0.20
**UVA**	1.64 ± 0.10 ^x,y^	2.12 ± 0.12 ^x,y^	5.22 ± 0.20 ^x,y^	5.86 ± 0.32 ^x,y^
**UVA+ CBD**	1.01 ± 0.07 ^z^	1.71 ± 0.14 ^z^	4.00 ± 0.18 ^y,z^	4.62± 0.20 ^z^
**UVB**	1.42 ± 0.10 ^y^	2.04 ± 0.15 ^x,y^	5.05 ± 0.19 ^x,y^	5.52 ± 0.38 ^x,y^
**UVB + CBD**	1.00 ± 0.07 ^z^	1.31 ± 0.15 ^z^	3.67 ± 0.26 ^z^	4.26± 0.22^z^
**Ps**	**0.95 ± 0.14 ^a^**	**1.37 ± 0.14 ^a^**	**2.24 ± 0.19 ^a^**	**3.27 ± 0.20 ^a^**
**Ps + CBD**	0.84 ± 0.10	1.29 ± 0.10	1.85 ± 0.20	2.57 ± 0.25
**Ps + UVA**	1.02 ± 0.12	1.46 ± 0.12	3.21 ± 0.26	3.83 ± 0.29 ^y^
**Ps + UVA + CBD**	1.05 ± 0.10	1.62 ± 0.13 ^x^	3.65 ± 0.23 ^x,y^	4.22 ± 0.21 ^x,y^
**Ps + UVB**	1.01 ± 0.10	1.44 ± 0.13	3.12 ± 0.23	3.73 ± 0.23
**Ps + UVB + CBD**	1.03 ± 0.16	1.63 ± 0.14 ^x^	4.85 ± 0.25 ^x,y,z^	3.99 ± 0.20 ^x,y^

PI—phosphatidylinositol; PS—phosphatidylserine; PE—phosphatidylethanolamine; PC—phosphatidylcholine; ^a^—differences between Ps and Ctr groups; ^x^—differences vs. Ctr or Ps group; ^y^—differences vs. CBD treated groups [CBD and Ps + CBD]; ^z^—differences vs. UVA or UVB treated groups [UVA/UVB and Ps + UVA/UVB].

**Table 2:** Phospholipids membrane composition of fibroblasts isolated from skin of healthy subjects (Ctr) (*n* = 6) and psoriatic patients (Ps) (*n* = 6) exposed to UVA (30 J/cm^2^) and UVB (60 mJ/cm^2^) radiation as well as untreated and treated with CBD (4 μM). The mean values ± SD and statistically significant differences for *p* < 0.05 are presented;

Groups	PI	PS	PE	PC
	µg/mg Protein			
**Control**	**1.69 ± 0.12**	**2.72 ± 0.17**	**5.88 ± 0.25**	**6.99 ± 0.30**
**CBD**	1.55 ± 0.11	2.40 ± 0.10	5.02 ± 0.26	6.09 ± 0.23
**UVA**	3.55 ± 0.31 ^x,y^	4.52 ± 0.26 ^x,y^	10.12 ± 0.38 ^x,y^	12.24 ± 0.81 ^x,y^
**UVA+ CBD**	2.94 ± 0.20 ^y^	4.04 ± 0.21 ^y^	6.24 ± 0.30 ^z^	7.39 ± 0.30 ^z^
**UVB**	3.10 ± 0.19 ^x,y^	4.42 ± 0.22 ^x,y^	9.05 ± 0.35 ^x,y^	11.35 ± 0.41 ^x,y^
**UVB + CBD**	2.51 ± 0.20	2.89 ± 0.15 ^z^	6.01 ± 0.33 ^z^	7.24± 0.35 ^z^
**Ps**	**1.20 ± 0.10 ^a^**	**2.08 ± 0.22 ^a^**	**4.60 ± 0.29 ^a^**	**5.32 ± 0.26 ^a^**
**Ps + CBD**	0.94 ± 0.10	1.66 ± 0.19	3.84 ± 0.22	4.40 ± 0.25
**Ps + UVA**	1.58 ± 0.11 ^x^	2.26 ± 0.21	5.31 ± 0.28 ^x,y^	6.22 ± 0.33 ^x,y^
**Ps + UVA + CBD**	1.64 ± 0.12 ^x,y^	2.52 ± 0.18 ^x,y^	5.33 ± 0.32 ^x,y^	6.39 ± 0.36 ^x,y^
**Ps + UVB**	1.66 ± 0.15 ^x,y^	2.37 ± 0.21	5.25 ± 0.25 ^y^	6.17 ± 0.33 ^x,y^
**Ps + UVB +CBD CBD**	1.84 ± 0.18 ^x,y^	3.66 ± 0.23 ^x,y^	5.46 ± 0.30 ^x,y^	6.37 ± 0.28 ^x,y^

^a^—differences between Ps and Ctr groups; ^x^—differences vs. Ctr or Ps group; ^y^—differences vs. CBD treated groups [CBD and Ps + CBD]; ^z^—differences vs. UVA or UVB treated groups [UVA/UVB and Ps + UVA/UVB].

**Table 3:** The value *C_TA_*_,_ *C_TB_*_,_ *K_AH_*, *K_BOH_* of keratinocytes and fibroblasts isolated from skin of healthy subjects (Ctr) (*n* = 6) a psoriatic patients (Ps) (*n* = 6) exposed to UVA (30 J/cm^2^) and UVB (60 mJ/cm^2^) radiation as well as untreated and treated with CBD (4 μM). The mean values ± SD and statistically significant differences for *p* < 0.05 are presented. ^a^—differences between Ps and Ctr groups; ^x^—differences vs. Ctr or Ps group; ^y^—differences vs. CBD treated groups [CBD and Ps + CBD]; ^z^—differences vs. UVA or UVB treated groups [UVA/UVB and Ps + UVA/UVB].

Cells/Groups	Parameters			
	*C_TA_* [10^−6^ mol/m^2^]	*C_TB_* [10^−6^ mol/m^2^]	*K_AH_* [10^2^ m^3^/mol]	*K_BOH_* [10^7^ m^3^/mol]
**Keratinocytes**				
**Ctr**	**3.04 ± 0.09**	**0.55 ± 0.05**	**1.85 ± 0.06**	**3.86 ± 0.09**
**CBD**	3.31 ± 0.11 ^x^	0.57 ± 0.04	1.78 ± 0.05	4.09 ± 0.08
**UVA**	3.99 ± 0.12 ^x,y^	0.63 ± 0.05	0.88 ± 0.04 ^x,y^	5.65 ± 0.10 ^x,y^
**UVA + CBD**	3.65 ± 0.08 ^x,z^	0.50 ± 0.04	1.18 ± 0.05 ^z^	4.26 ± 0.08 ^z^
**UVB**	3.96 ± 0.15 ^x,y^	0.59 ± 0.05	0.78 ± 0.04 ^x,y^	6.50 ± 0.11 ^x,y^
**UVB + CBD**	3.40 ± 0.07 ^z^	0.75 ± 0.04 ^x,y^	1.10 ± 0.06 ^z^	4.60 ± 0.11 ^z^
**Ps**	**4.89 ± 0.11 ^a^**	**1.38 ± 0.06 ^a^**	**0.88 ± 0.07 ^a^**	**6.16 ± 0.11 ^a^**
**Ps + CBD**	3.42 ± 0.10 ^x^	0.95 ± 0.05	1.68 ± 0.07 ^x^	3.02 ± 0.08 ^x^
**Ps + UVA**	3.96 ± 0.12 ^x,y^	1.69 ± 0.08 ^x,y^	1.29 ± 0.05 ^y^	5.15 ± 0.14 ^y^
**Ps + UVA + CBD**	3.91 ± 0.07 ^x,y^	1.34 ± 0.06 ^z^	1.37 ± 0.05 ^x^	4.99 ± 0.08 ^x,y^
**Ps + UVB**	3.93 ± 0.10 ^x,y^	0.85 ± 0.07 ^x^	1.01 ± 0.06 ^y^	5.04 ± 0.10 ^y^
**Ps + UVB + CBD**	3.49 ± 0.09 ^x,z^	0.92 ± 0.06	1.38 ± 0.05 ^x,z^	4.37 ± 0.09 ^x,z^
**Fibroblasts**				
**Ctr**	**3.42 ± 0.10**	**0.37 ± 0.04**	**2.88 ± 0.06**	**3.78 ± 0.05**
**CBD**	3.39 ± 0.08	0.56 ± 0.04 ^x^	3.48 ± 0.05 ^x^	3.78 ± 0.06
**UVA**	4.09 ± 0.11 ^x,y^	1.58 ± 0.07 ^x^	1.83 ± 0.04 ^x,y^	5.25 ± 0.07 ^x,y^
**UVA+ CBD**	3.64 ± 0.09 ^z^	1.07 ± 0.04 ^x,z^	0.98 ± 0.04 ^x,y,z^	4.29 ± 0.06 ^z^
**UVB**	3.92 ± 0.11 ^x,y^	1.84 ± 0.08 ^x^	0.88 ± 0.04 ^x,y^	5.67 ± 0.07 ^x,y^
**UVB + CBD**	3.52 ± 0.10 ^z^	1.19 ± 0.06 ^x,z^	1.04 ± 0.04 ^x,y^	4.52 ± 0.07 ^z^
**Ps**	**5.80 ± 0.09 ^a^**	**0.75 ± 0.05 ^a^**	**0.58 ± 0.03 ^a^**	**7.81 ± 0.12 ^a^**
**Ps + CBD**	3.38 ± 0.07 ^x^	0.88 ± 0.06	0.88 ± 0.05	4.19 ± 0.10 ^x^
**Ps + UVA**	3.93 ± 0.08 ^x,y^	0.85 ± 0.04	0.78 ± 0.04	5.24 ± 0.08 ^x^
**Ps + UVA + CBD**	3.24 ± 0.06 ^x,z^	0.55 ± 0.04 ^x,z^	0.98 ± 0.04 ^x^	4.26 ± 0.08 ^x,z^
**Ps + UVB**	4.35 ± 0.08 ^x^	1.06 ± 0.08 ^x^	1.08 ± 0.06 ^x^	4.67 ± 0.08 ^x^
**Ps + UVB + CBD**	3.20 ± 0.07 ^x,z^	0.56 ± 0.04 ^y,z^	0.96 ± 0.04 ^x^	4.28 ± 0.08 ^x^

## Data Availability

Data available on request.

## References

[1] Kendall A.C., Nicolaou A. (2013). Bioactive lipid mediators in skin inflammation and immunity. Prog. Lipid Res..

[2] Muallem S., Young W., Jha C.A., Ahuja M. (2017). Lipids at membrane contact sites: Cell signaling andion transport. EMBO Rep..

[3] Dobrzyńska I., Szachowicz-Petelska B., Pędzińska-Betiuk A., Figaszewski Z.A., Skrzydlewska E. (2020). Effects of hypertension and FAAH inhibitor treatment of rats with primary and secondary hypertension considering the physicochemical properties of erythrocytes. Toxicol. Mech. Method..

[4] Dobrzyńska I., Gęgotek A., Gajko E., Skrzydlewska E., Figaszewski Z.A. (2018). Effects of rutin on the physicochemical properties of skin fibroblasts membrane disruption following UV radiation. Chem. Biol. Interact..

[5] Jarocka-Karpowicz I., Biernacki M., Wroński A., Gęgotek A., Skrzydlewska E. (2020). Cannabidiol effects on phospholipid metabolism in keratinocytes from patients with psoriasis vulgaris. Biomolecules.

[6] Kemeny L., Varga E., Novak Z. (2019). Advances in phototherapy for psoriasis and atopic dermatitis. Expet. Rev. Clin. Immunol..

[7] Dang L., Wang Y., Xue Y., He L., Li Y., Xiong J. (2015). Low-dose UVB irradiation prevents MMP2-induced skin hyperplasia by inhibiting inflammation and ROS. Oncol. Rep..

[8] Gęgotek A., Biernacki M., Ambrożewicz E., Surażyński A., Wroński A., Skrzydlewska E. (2016). The cross-talk between electrophiles, antioxidant defence and the endocannabinoid system in fibroblasts and keratinocytes after UVA and UVB irradiation. J. Dermatol. Sci..

[9] Ambrożewicz E., Wójcik P., Wroński A., Łuczaj W., Jastrząb A., Žarković N., Skrzydlewska E. (2018). Pathophysiological alterations of redox signaling and endocannabinoid system in granulocytes and plasma of psoriatic patients. Cells.

[10] Rácz E., Prens E.P. (2017). Phototherapy of Psoriasis, a Chronic Inflammatory Skin Disease. Adv. Exp. Med. Biol..

[11] Lee C.H., Wu S.B., Hong C.H., Yu H.S., Wei Y.H. (2013). Molecular mechanism of UV-induced apoptosis and its effects on skin residential cells: The implication in UV-based phototherapy. Int. J. Mol. Sci..

[12] Atalay S., Jarocka-Karpowicz I., Skrzydlewska E. (2020). Antioxidative and anti-Inflammatory properties of cannabidiol. Antioxidants.

[13] Morales P., Reggio P.H., Jagerovic N. (2017). An overview on medicinal chemistry of synthetic and natural derivatives of cannabidiol. Front. Pharmacol..

[14] Atalay S., Dobrzyńska I., Gęgotek A., Skrzydlewska E. (2020). Cannabidiol protects keratinocyte cell membranes following exposure to UVB and hydrogen peroxide. Redox Biol..

[15] Pan H., Mukhopadhyay P., Rajesh M., Patel V., Mukhopadhyay B., Gao B., Hasko G., Pacher P. (2009). Cannabidiol attenuates cisplatin-induced nephrotoxicity by decreasing oxidative/nitrosative stress, inflammation, and cell death. J. Pharmacol. Exp. Ther..

[16] Gęgotek A., Atalay S., Domingues P., Skrzydlewska E. (2019). The Differences in the Proteome Profile of cannabidiol-treated skin fibroblasts following UVA or UVB Irradiation in 2D and 3D cell cultures. Cells.

[17] Vallée A., Lecarpentier Y., Guillevin R., Vallée J.N. (2017). Effects of cannabidiol interactions withWnt/β-catenin pathway and PPARγon oxidative stress and neuroinflammation in Alzheimer’s disease. Acta Biochim. Biophys. Sin..

[18] Casares L., García V., Garrido-Rodríguez M., Millán E., Collado J.A., García-Martín A., Peñarando J., Calzado M.A., de la Vega L., Muñoz E. (2020). Cannabidiol induces antioxidant pathways in keratinocytes by targeting BACH1. Redox Biol..

[19] Jastrząb A., Gęgotek A., Skrzydlewska E. (2019). Cannabidiol regulates the expression of keratinocyte proteins involved in the inflammation process through transcriptional regulation. Cells.

[20] Fotakis G., Timbrell J.A. (2006). In vitro cytotoxicity assays: Comparison of LDH, neutral red, MTT and protein assay in hepatoma cell lines following exposure to cadmium chloride. Toxicol. Lett..

[21] Folch J., Lees M., Stanley G.H.S. (1957). A simple method for the isolation and purification of total lipids from animal tissues. J. Biol. Chem..

[22] Dobrzyńska I., Szachowicz-Petelska B., Darewicz B., Figaszewski Z.A. (2015). Characterization of human bladder cell membrane during cancer transformation. J. Membr. Biol..

[23] Jourdian G.W., Dean L., Roseman S. (1971). The sialic acids. XI. A periodate-resorcinol method for the quantitative estimation of free sialic acids and their glycosides. J. Biol. Chem..

[24] Luo X.P., Yazdanpanah M., Bhooi N., Lehotay D.C. (1995). Determination of aldehydes and other lipid peroxidation products in biological samples by gas chromatography-mass spectrometry. Anal. Biochem..

[25] Gęgotek A., Bielawska K., Biernacki M., Dobrzyńska I., Skrzydlewska E. (2017). Time-dependent effect of rutin on skin fibroblasts membrane disruption following UV radiation. Redox Biol..

[26] Dobrzyńska I., Szachowicz-Petelska B., Weresa J., Figaszewski Z.A., Skrzydlewska E. (2019). Changes in physicochemical properties of kidney cells membrane as a consequence of hypertension and treatment of hypertensive rats with FAAH inhibitor. Chem. Biol. Interact..

[27] Dobrzyńska I., Skrzydlewska E., Figaszewski Z.A. (2006). Parameters characterizing acid-base equilibria between cell membrane and solution and their application to monitoring the effect of various factors on the membrane. Bioelectrochemistry.

[28] Kadam D.P., Suryakar A.N., Ankush R.D., Kadam C.Y., Deshpande K.H. (2010). Role of oxidative stress in various stages of psoriasis. Indian J. Clin. Biochem..

[29] Ozcan A., Ogun M., Gowder S.J.T. (2015). Biochemistry of reactive oxygen and nitrogen species. Basic Principles and Clinical Significance of Oxidative Stress.

[30] Dobrzyńska I., Szachowicz-Petelska B., Wroński A., Jarocka-Karpowicz I., Skrzydlewska E. (2020). Changes in the physicochemical properties of blood and skin cell membranes as a result of psoriasis vulgaris and psoriatic arthritis development. Int. J. Mol. Sci..

[31] Lubbers J., Rodriguez E., van Kooyk Y. (2018). Modulation of immune tolerance via Siglec-sialic acid interactions. Front. Immunol..

[32] Ghosh S., Ghosh S. (2020). Sialic Acid and Biology of Life: An Introduction. Sialic Acid and Sialoglycoconjugates in the Biology of Life, Health and Disaease.

[33] Łuczaj W., Dobrzyńska I., Wroński A., Domingues M.R., Domingues P., Skrzydlewska E. (2020). Cannabidiol-mediated changes to the phospholipid profile of UVB-irradiated keratinocytes from psoriatic patients. Int. J. Mol. Sci..

[34] Kim W.B., Jerome D., Yeung J. (2017). Diagnosis and management of psoriasis. Can. Fam. Physician.

[35] Gęgotek A., Bielawska K., Biernacki M., Zaręba I., Surażyński A., Skrzydlewska E. (2017). Comparison of protective effect of ascorbic acid on redox and endocannabinoid systems interactions in in vitro cultured human skin fibroblasts exposed to UV radiation and hydrogen peroxide. Arch. Dermatol. Res..

[36] Juzeniene A., Moan J. (2012). Beneficial effects of UV radiation other than via vitamin D production. Derm. Endocrinol..

[37] Wäster P.K., Ollinger K.M. (2009). Redox-dependent translocation of p53 to mitochondria or nucleus in human melanocytes after UVA- and UVB-induced apoptosis. J. Investig. Dermatol..

[38] Gruber F., Wondrak G.T. (2016). The skin lipidome under environmental stress-technological platforms, molecular pathways and translational opportunities. Skin Stress Response Pathways.

[39] Dalmau N., Andrieu-Abadie N., Tauler R., Bedia C. (2018). Phenotypic and lipidomic characterization of primary human epidermal keratinocytes exposed to simulated solar UV radiation. J. Dermatol. Sci..

[40] Olivier E., Dutot M., Regazzetti A., Dargere D., Auzeil N., Laprevote O., Szczur P. (2017). Lipid deregulation in UV irradiated skin cells: Role of 25-hydroxycholesterol in keratinocyte differentiation during photoaging. J. Steroid Biochem. Mol. Biol..

[41] Zeidan Y.H., Wu B.X., Jenkins R.W., Obeid L.M., Hannun Y.A. (2008). A novel role for protein kinase Cδ-mediated phosphorylation of acid sphingomyelinase in UV light-induced mitochondrial injury. FASEB J..

[42] Cha H.J., He C., Zhao H., Dong Y., An I.-S., Kim Y.J. (2016). Intercellular and intracellular functions of ceramides and their metabolites in skin (Review). Int. J. Mol. Med..

[43] Ramírez de Molina A., Gallego-Ortega D., Sarmentero-Estrada J., Lagares D., Gómez del Pulgar T., Bandrés E., García-Foncillas J., Lacal J.C. (2008). Choline kinase as a link connecting phospholipid metabolism and cell cycle regulation: Implications in cancer therapy. Int. J. Biochem. Cell Biol..

[44] Gęgotek A., Domingues P., Wroński A., Ambrożewicz E., Skrzydlewska E. (2019). The Proteomic Profile of Keratinocytes and Lymphocytes in Psoriatic Patients. Proteom. Clin. Appl..

[45] Cannavò S.P., Riso G., Casciaro M., Di Salvo E., Gangemi S. (2019). Oxidative stress involvement in psoriasis: A systematic review. Free Radic. Res..

[46] Ren Y., Liu X., Geng R., Lu Q., Rao R., Tan X., Yang X., Liu W. (2018). Increased Level of α2,6-Sialylated Glycans on HaCaT Cells Induced by Titanium Dioxide Nanoparticles under UV Radiation. Nanomaterials.

[47] Wang Y., Zhang G.Y., Han Q.L., Wang J., Li Y., Yu C.H., Li Y.R., Yi Z.C. (2014). Phenolic metabolites of benzene induced caspase-dependent cytotoxicities to K562 cells accompanied with decrease in cell surface sialic acids. Environ. Toxicol..

[48] Suzuki O., Abe M. (2014). Galectin-1-mediated cell adhesion, invasion and cell death in human anaplastic large cell lymphoma: Regulatory roles of cell surface glycans. Int. J. Oncol..

[49] Weatherhead S.C., Farr P.M., Jamieson D., Hallinan J.S., Lloyd J.J., Wipat A., Reynolds N.J. (2011). Keratinocyte apoptosis in epidermal remodeling and clearance of psoriasis induced by UV radiation. J. Investig. Dermatol..

[50] Meesmann H.M., Fehr E.-M., Kierschke S., Herrmann M., Bilyy R., Heyder P., Blank N., Krienke S., Lorenz H.-M., Schiller M. (2010). Decrease of sialic acid residues as an eat-me signal on the surface of apoptotic lymphocytes. J. Cell Sci..

[51] Shenoy C., Shantaram M., Sharanya K., Shenoy M.M. (2015). Lipid-bound sialic acid in psoriasis and its correlation with disease severity. Saudi J. Health Sci..

[52] Rajendinar K.S., Ananthanarayanan R.H., Satheesh S., Rajappa M. (2014). Elevated levels of serum sialic acid and high-sensitivity C-reactive protein: Markers of systemic inflammation in patients with chronic heart failure. Br. J. Biomed. Sci..

[53] Kleiser S., Nyström A. (2020). Interplay between cell-surface receptors and extracellular matrix in skin. Biomolecules.

[54] Jellusova J., Nitschke L. (2012). Regulation of B cell functions by the sialic acid-binding receptors Siglec-G and CD22. Front. Immunol..

[55] Scheau C., Badarau I.A., Mihai G.L., Scheau A.-E., Costache D.O., Constantin C., Calina D., Caruntu C., Costache R.S., Caruntu A. (2020). Cannabinoids in the Pathophysiology of Skin Inflammation. Molecules.

[56] Bruni N., Della Pepa C., Oliaro-Bosso S., Pessione E., Gastaldi D., Dosio F. (2018). Cannabinoid delivery systems for pain and inflammation treatment. Molecules.

[57] Matsura T. (2014). Oxidized phosphatidylserine: Production and bioactivities. Yonago Acta Med..

[58] Chaurio R.A., Janko C., Muñoz L.E., Frey B., Herrmann M., Gaipl U.S. (2009). Phospholipids: Key players in apoptosis and immune regulation. Molecules.

[59] Kagan V.E., Bayır H., Tyurina Y.Y., Bolevich S.B., Maguire J.J., Fadeel B., Balasubramanian K. (2017). Elimination of the unnecessary: Intra- and extracellular signaling by anionic phospholipids. Biochem. Biophys. Res. Commun..

[60] Dobrzyńska I., Kotynska J., Szachowicz-Petelska B., Figaszewski Z.A. (2017). Determination of association constants of monovalent ions to sphingomyelin and phosphatidylinositol liposomal membranes by microelectrophoresis. Soft Mater..

[61] Kotynska J., Dobrzyńska I., Figaszewski Z.A. (2017). Association of alkali metal cations with phosphatidylcholine liposomal membrane surface. Eur. Biophys. J..

[62] Montone C.M., Cerrato A., Botta B., Cannazza G., Capriotti A.L., Cavaliere C., Citti C., Ghirga F., Piovesana S., Lagana A. (2020). Improved identification of phytocannabinoids using a dedicated structure-based workflow. Talanta.

